# SABCS 2017: lifestyle factors, hormone receptor-positive advanced disease, liquid biopsies, and prognosis

**DOI:** 10.1007/s12254-018-0433-x

**Published:** 2018-08-21

**Authors:** Gabriel Rinnerthaler, Simon Peter Gampenrieder, Richard Greil

**Affiliations:** 1 IIIrd Medical Department with Hematology and Medical Oncology, Oncologic Center, Paracelsus Medical University Salzburg, Müllner Hauptstraße 48, 5020 Salzburg, Austria; 2 Laboratory of Immunological and Molecular Cancer Research and Center for Clinical Cancer and Immunology Trials, Salzburg Cancer Research Institute, Salzburg, Austria; 3 Cancer Cluster Salzburg, Salzburg, Austria

**Keywords:** San Antonio Breast Cancer conference, CTS5, MONALEESA-7, PALOMA-2, ABCSG-34

## Abstract

This article reviews the clinically most relevant presentations at the San Antonio Breast Cancer Symposium (SABCS) 2017 on the topics lifestyle factors, hormone receptor-positive advanced disease, liquid biopsies, and prognosis.

In a retrospective analysis of the Women’s Health Initiative Observational Study, a reduction in the body mass index (BMI) of at least 5% within 3 years significantly reduced the risk of breast cancer compared to women with a stable weight (HR 0.77; 95% CI 0.78–0.98). In the MONALEESA-7 trial investigating ribociclib or placebo in combination with endocrine therapy as first-line treatment in pre- and perimenopausal women with hormone receptor-positive, human epidermal growth factor 2 (HER2)-negative metastatic breast cancer, a significantly longer progression-free survival was shown for patients treated with ribociclib compared to the placebo group (23.8 vs. 13.0 months; HR 0.55; 95% CI 0.43–0.72; *P* < 0.001). In a pooled toxicity and efficacy analysis of elderly women treated with a cyclin-dependent kinase 4/6 (CDK4/6) inhibitor in combination with an aromatase inhibitor in first-line, toxicities of higher grade were more common in elderly compared to younger patients, despite comparable efficacy. And the Clinical Treatment Score post-5 years (CTS5), accurately estimated the risk of late recurrence after 5 years of adjuvant endocrine treatment using routinely available clinical parameters.

## Review

Every year, the San Antonio Breast Cancer Conference (SABCS) condenses high-level breast cancer research. In this short review, the clinically most important presentations in the field of lifestyle factors, hormone receptor-positive advanced disease, liquid biopsies, and prognosis will be discussed.

### Lifestyle factors

Obesity is an established risk factor for the development of breast cancer in postmenopausal woman [1], whereas the influence of weight loss on breast cancer risk is not as clear, because data were conflicting [2]. At the San Antonio Breast Cancer Conference (SABCS) 2017, Chlebowski R. T. presented a retrospective analysis of the associations between weight change and breast cancer incidence in postmenopausal women participating in the Women’s Health Initiative Observational Study [3]. During a median follow-up of 11.4 years, 3061 of the 61,335 women enrolled in the study developed invasive breast cancer. Height and weight were measured at baseline and at year 3. Compared to a stable body mass index (BMI), weight loss of ≥ 5% within 3 years statistically decreased breast cancer risk in multivariate analysis by 12% (hazard ratio [HR] 0.88; 95% CI 0.78–0.98), irrespective of whether weight loss was intentional or unintentional. Weight loss of ≥ 15% of body weight was associated with a 37% reduction in breast cancer risk. Contrarily, women with weight gain had a 50% increased risk of triple-negative breast cancer (HR 1.54 95% CI 1.16–2.05), but the overall breast cancer risk was comparable to women with stable weight (Table 1).

**Table 1:** Women’s Health Initiative (WHI) Observational Study—Weight Loss and Breast Cancer Incidence in Postmenopausal Women [4]

Percentage weight change between baseline and year 3	Cancer cases	HR (95% CI) multivariable adjusted^d^
Stable weight (within ±5%)	2092	Reference
Weight gain (>5%)^a^	620	1.02 (0.93–1.11)
**Weight loss (>5%)** ^**b**^	349	**0.88 (0.78–0.98)**
Intentional^c^	229	0.91 (0.79–1.04)
Unintentional^c^	120	0.82 (0.68–0.99)

^a^Women with weight gain had a significantly higher incidence of TNBC (triple-negative breast cancer; HR 1.54 95% CI 1.16–2.05)

^b^Adjustment for mammography frequency did not alter findings (HR 0.88 95% CI 0.78–0.99)

^c^Statistical test between intentional and unintentional weight loss groups found no significant difference (*P* = 0.2)

^d^Adjusted for the Breast Cancer Risk Assessment Tool (BCRAT) score (includes age at enrollment, race/ethnicity, age of menarche, age of the mother at the birth of her first live child, number of first-degree relatives with breast cancer, and the number of previous breast biopsies), education, smoking pack-years, recreational physical activity, alcohol consumption, history of hormone therapy use, parity, and body mass index (BMI)

### Advanced hormone receptor-positive breast cancer

In San Antonio, the first results of the MONALEESA-7 study were presented [5]. In this randomized phase III trial, ribociclib or placebo, each combined with goserelin and tamoxifen or a non-steroidal aromatase inhibitor, was investigated as first-line treatment in 627 pre- and perimenopausal women with hormone receptor (HR)-positive, human epidermal growth factor 2(HER2)-negative advanced breast cancer (ABC). Pretreatment with one line of palliative chemotherapy was allowed. Patients treated with the cyclin dependent kinase 4/6 (CDK4/6) inhibitor ribociclib had a significantly longer progression-free survival (PFS), the primary endpoint of the trial, compared to the placebo group with 23.8 versus 13.0 months (HR 0.55; 95% CI 0.43–0.72; *P* < 0.001). This effect could be shown in all predefined subgroups, irrespective of the endocrine combination partner. The overall response rate (ORR) was also in favor of the ribociclib arm (51 vs. 36%). Furthermore, patient-reported outcomes of this study were presented. The time from start of treatment to deterioration in global health status scale of at least 10% (assessed with the breast cancer-specific quality of life questionnaire EORTC QLQ-30) was statistically significantly longer in the ribociclib group compared to the placebo arm (HR 0.70; 95% CI 0.53–0.92; *P* < 0.004). The toxicity profile was in line with the previously published MONALEESA-2 and MONALEESA-3 trial results [6, 7]. Neutropenia grade ≥3 and febrile neutropenia occurred in 60.6 and 2.1% of patients in the ribociclib group and in 3.6 and 0.6% in the control group, respectively. QT time prolongation was detected in 6.9% in the ribociclib arm versus 1.2% in the placebo arm. QT prolongation events were, however, not associated with clinical symptoms or arrhythmia.

A pooled toxicity and efficacy analysis of elderly women treated with a CDK4/6 inhibitor in combination with an aromatase inhibitor in first-line registration trials was presented by the American Food and Drug Administration (FDA; [8]). No efficacy difference was detected across age subgroups, irrespective of age thresholds. Adverse events (AEs) of higher grade, however, were more common in elderly compared to younger patients (grade 3–4 AE: age <66 years: 66%; age >65 years: 80%; age >70 years: 82%; Table 2). The discontinuation rate was twice as high in patients older than 65 years (16%) compared to women younger than 65 years (8%).

**Table 2:** Pooled adverse events in registration trials of CDK4/6 inhibitors in combination with an aromatase inhibitor as first-line treatment in hormone receptor-positive, HER2-negative breast cancer patients [8]

	Age < 65 years *N* = 625 (%)	Age ≥ 65 years *N* = 479 (%)	Age ≥ 70 years *N* = 280 (%)
AE leading to dose reduction and/or interruption	411 (66)	360 (75)	216 (77)
AE leading to discontinuation	50 (8)	76 (16)	48 (17)
Grade 1–2 AEs	610 (98)	470 (98)	277 (99)
Grade 3–4 AEs	417 (66)	385 (80)	229 (82)
Grade 5 AEs	7 (1)	11 (2)	8 (3)
Serious AEs	103 (16)	147 (31)	93 (33)

*AE* adverse events

In a retrospective analysis of the PALOMA-2 trial, a phase III trial investigating palbociclib or placebo, each in combination with letrozole, in postmenopausal women with HR-positive, HER2-negative ABC, PFS was correlated with intrinsic subtypes defined by the PAM50 algorithm [9]. Both patients with luminal A and luminal B subtypes had a longer PFS when treated with palbociclib compared to the placebo arm (luminal A: 11.0 vs. 19.6; luminal B 17.0 vs. 30.4 months). Interestingly, in patients with non-luminal breast cancer subtypes, no benefit from the addition of palbociclib to endocrine treatment was seen.

### Circulating tumor cells and liquid biopsy

Several studies showed that the presence and burden of circulating tumor cells (CTS) is correlated with worse prognosis in early and advanced breast cancer patients [10–12]. In a retrospective analysis of a phase III trial investigating the efficacy of adjuvant bevacizumab in early HER2-negative breast cancer, CTC burden measurement approximately 5 years after diagnosis at a single timepoint was correlated with the late recurrence risk [13]. In patients with HR-positive cancers, the presence of CTC was associated with a worse prognosis (HR 21.7, *P* < 0.001). In triple-negative cancer (TNBC), the detection of CTC had no impact on prognosis and only few late recurrences were detected in the TNBC group. Mayor limitations of this analysis are a short median follow-up (1.8 years in HR-positive patients) and CTC measurement at a single timepoint only.

Somatic mutations are relatively rare in TNBC, with an average of seven driver mutations per primary tumor [4]. In contrast, somatic copy number alterations (SCNAs) are frequent, with more than 50% genome alteration in most primary TNBC tumors [14]. In an already fully published work, a genome-wide copy number analysis of chemotherapy-resistant metastatic TNBC was performed from cell-free DNA [15]. Using a special cost-effective sequencing technique called ultra-low-pass whole-genome sequencing, the tumor fraction (TF) was calculated based on the detection of SCNAs. TF was an independent prognostic factor in this TNBC cohort. In an additionally reported case of a patient with metastatic TNBC, a correlation between reduction and gain of TF and treatment response or progression was nicely illustrated.

### Prognosis and prediction

As previously published by the Early Breast Cancer Trialists’ Collaborative Group (EBCTCG), about 10 to 40% of HR-positive breast cancer patients suffer from late recurrences, depending on nodal status, tumor size, and grading [16]. At SABCS 2017, Ivanva Sestak presented a prognostic score, the Clinical Treatment Score post-5 years (CTS5), estimating the risk of disease recurrence after 5 years of endocrine treatment [17]. This score was developed in the ATAC (Arimidex, Tamoxifen, Alone or in Combination) trial dataset (*N* = 4735) and validated in the Breast International Group (BIG) 1–98 trial dataset (*N* = 6711). CTS5 integrates the number of affected lymph nodes, tumor size, age, and tumor grading. In both datasets, the CTS5 was able to clearly separate low- (defined in the training cohort as <5% recurrence risk), intermediate- (5–10%), and high-risk (>10%) groups for late recurrences 5 to 10 years after diagnosis. In the validation cohort, 43% of patients were classified as low risk, with an observed disease recurrence rate of 3.6% (95% CI 2.7 to 4.9%) during years 5 to 10 (Table 3). Recently, the final CTC5 algorithm, derived from pooled data of both data sets, was fully published. [18].

**Table 3:** Clinical Treatment Score post-5 years (CTS5): estimation of disease recurrence 5 to 10 years after diagnosis of hormone receptor-positive breast cancer patients treated with 5 years of endocrine treatment

Risk groups	5- to 10-year disease recurrence risk (%)	95% CI (%)	No. of events
ATAC dataset (*N* = 4735)			
Low	2.5	1.8–3.4	40
Intermediate	7.7	6.3–9.5	95
High	20.3	17.2–24	195
BIG 1–98 dataset (*N* = 6711)			
Low	3.6	2.7–4.9	64
Intermediate	6.9	5.6–8.5	108
High	17.3	14.8–20.1	198

In the randomized phase II Austrian Breast and Colorectal Cancer Study Group (ABCSG) 34 trial, the efficacy of a mucin-1 (MUC1) vaccination in combination with neoadjuvant treatment was tested in HER2-negative breast cancer patients. Based on prognostic factors, patients were treated with neoadjuvant endocrine treatment or with neoadjuvant chemotherapy. At SABCS 2017, Peter Dubsky presented a translational analysis of the ABCSG 34 trial [19] investigating the correlation of residual cancer burden (RCB) index, a quantification method of residual disease in the surgical resection specimens [20], and the risk category given by the gene expression test EndoPredict® (Myriad Genetics Inc., Salt Lake City, Utah, USA). In patients with hormone receptor-positive disease treated with neoadjuvant endocrine therapy, a high genomic risk was associated with a lower chance of treatment response (negative predicting value, NPV, 92%). In concordance, a lower genomic risk was associated with worse response to treatment (NPV 100%) in patients treated with neoadjuvant chemotherapy, suggesting that chemotherapy could be spared in case of EndoPredict® low risk.

## Conclusion

Some of the reviewed presentations will influence our daily clinical practice. The retrospective analysis of the Women’s Health Initiative Observational Study emphasizes the importance of lifestyle modifications for breast cancer prevention. In MONALEESA-7, the efficacy of CDK4/6 inhibition as first-line treatment also in pre- and perimenopausal women with HR-positive, HER2-negative ABC was clearly shown, and this treatment should be considered as a new standard of care. A careful and close monitoring of elderly patients under treatment with CDK4/6 inhibitors is implicated by the pooled analysis of elderly patients treated in first-line registration trials with CDK4/6 inhibitors. Routinely available parameters included in the CTS5 risk score support physicians in identifying patients with a higher risk of late recurrence and who may be candidates for an extended adjuvant endocrine treatment beyond 5 years. Finally, EndoPredict® may help to guide neoadjuvant therapy in patients with hormone receptor-positive, HER2-negative early breast cancer.
